# Molecular Epidemiology of Foot-and-Mouth Disease Virus in the Context of Transboundary Animal Movement in the Far North Region of Cameroon

**DOI:** 10.3389/fvets.2018.00320

**Published:** 2018-12-14

**Authors:** Miranda R. Bertram, Carla Bravo de Rueda, Rebecca Garabed, Simon Dickmu Jumbo, Mark Moritz, Steven Pauszek, Souley Abdoulkadiri, Luis L. Rodriguez, Jonathan Arzt

**Affiliations:** ^1^Foreign Animal Disease Research Unit, Agricultural Research Service, Department of Agriculture, Plum Island Animal Disease Center (PIADC), Greenport, NY, United States; ^2^Research Participation Program, Plum Island Animal Disease Center, Oak Ridge Institute for Science and Education, Oak Ridge, TN, United States; ^3^Department of Veterinary Preventive Medicine, The Ohio State University, Columbus, OH, United States; ^4^Laboratoire National Vétérinaire (LANAVET), Garoua, Cameroon; ^5^Department of Anthropology, The Ohio State University, Columbus, OH, United States

**Keywords:** transboundary, cattle, FMDV, Cameroon, epidemiology, foot-and-mouth disease

## Abstract

Transboundary movement of animals is an important mechanism for foot-and-mouth disease virus (FMDV) spread in endemic regions, such as Cameroon. Several transboundary animal trade routes cross the Far North Region of Cameroon, and cattle moved on foot along these routes often come in contact with native (sedentary and transhumant) herds. The purpose of this study was to investigate the role of transboundary trade cattle in the epidemiology of FMDV in the Far North Region of Cameroon. A total of 582 oropharyngeal fluid (OPF) samples were collected from asymptomatic transboundary trade cattle at official border check points and 57 vesicle epithelial tissues were collected from clinically affected native cattle in the Far North Region of Cameroon during 2010–2014. Viral protein 1 (VP1) coding sequences were obtained from 6 OPF samples from transboundary cattle (4 serotype O, 2 serotype SAT2) and 19 epithelial tissue samples from native cattle (7 serotype O, 3 serotype SAT2, 9 serotype A). FMDV serotype O viruses belonged to two topotypes (East Africa-3 and West Africa), and phylogenetic analyses suggested a pattern of continuous transmission in the region. Serotype SAT2 viruses belonged to a single topotype (VII), and phylogenetic analysis suggested a pattern of repeated introductions of different SAT2 lineages in the region. Serotype A viruses belonged to topotype AFRICA/G-IV, and the pattern of transmission was unclear. Spearman rank correlation analysis of VP1 coding sequences obtained in this study from transboundary and native cattle showed a positive correlation between genetic distance and time for serotype O (ρ = 0.71, *p* = 0.003) and between genetic distance and geographic distance for serotype SAT2 (ρ = 0.54, *p* = 0.1). These data suggest that transboundary trade cattle participate in the transmission of FMDV in the Far North Region of Cameroon, however the dynamics and direction of transmission could not be determined in this study. Results of this study contribute to the understanding of transboundary FMDV epidemiology in Central Africa and will help to inform control programs in Cameroon and in the region.

## Introduction

Foot-and-mouth disease virus (FMDV; *Aphthovirus, Picornaviridae*) causes foot-and-mouth disease (FMD), one of the most economically important diseases of livestock worldwide (1). Acute infection is characterized by loss of appetite, fever, and formation of characteristic vesicles on the feet, udders, and in the oral cavity of cloven-hoofed animals (2, 3). Mortality is usually low, however high morbidity of the disease results in economic losses in endemic countries due to decreased production, regional quarantine practices, and trade restrictions (4). There are seven serotypes of FMDV: O, A, C, Asia-1, SAT1, SAT2, and SAT3; with multiple topotypes and genetic lineages within each serotype (5, 6). Seven endemic geographical regions, or pools, have been described, wherein countries within each pool share similar FMD viruses (7–9). Antigenic variation within a serotype necessitates that vaccines must be carefully matched to outbreak strains to ensure efficacy (10). For this reason, effective control of the disease is dependent on the ability to identify regional transmission patterns and predict which vaccine strains will protect the livestock population.

Transboundary animal movement has been shown to play a major role in FMD spread and introduction into FMD-free areas (8, 11). Transboundary movement has also been implicated in FMD outbreaks in endemic regions, such as South Asia and West Africa (12–15). In non-endemic countries, control of animal movement, including temporary cessation of transboundary trade of animals, has played a key role in eradication of FMD epidemics (11). However, limiting animal movement is not practical in endemic regions, where outbreaks occur regularly and are often unreported. Additionally, the practice of seasonal transhumance and movement of animals on foot are further challenges to the control of animal movements in Central Africa.

Serotypes O, A, SAT1, and SAT2 are endemic in the OIE-defined FMDV pool 5 (West and Central Africa), which includes Cameroon. FMDV serotypes O and A were first isolated from samples collected in Cameroon in 1931 and 1975, respectively (16, 17), and serotype SAT2 was first isolated in 2000 (18). However, sampling has been sporadic, and serotypes were likely circulating in the country prior to the first reports. Serologic evidence of serotypes SAT1 and SAT3 has also been reported in Cameroon in cattle (19), although the presence of SAT3 has not been confirmed. Based on serological data from 2010 used to reconstruct historic FMDV exposure in the Far North Region, serotypes O and SAT1 are likely maintained with continuous transmission within the population, whereas other serotypes likely require outside sources of reintroduction to explain the observed serological patterns (20). Additionally, phylogenetic analysis of FMDV strains from outbreaks in Cameroon in 2000–2001 and 2010–2012 showed serotype SAT2 strains were closely related to isolates from Eritrea, Saudi Arabia, and Libya, and serotypes A and O strains were closely related to historical samples from Cameroon (18, 19). These inferred patterns of transmission are consistent with patterns observed elsewhere in sub-Saharan Africa (21–23).

Livestock rearing is the primary economic activity in the northern regions of Cameroon (Adamawa, North, and Far North Regions), and the Far North contains about 2.25 million cattle (37.5% of the national herd) (24). The native cattle population in the Far North Region of Cameroon is a mixture of sedentary and transhumant animals (hereafter referred to as “native” cattle); these two groups share pasture and water resources and utilize the same domestic trade networks (within Cameroon) and veterinary services (19, 25). Additionally, animals are moved on foot along several transboundary animal trade routes crossing the Far North Region of Cameroon, originating from Chad and Sudan, with final destination markets in Nigeria (19). These unique movement patterns likely bring transboundary trade cattle into contact with native herds, and may provide a mechanism for virus spread into the Far North Region from external sources. However, the roles of transboundary and within-country animal movement in FMDV epidemiology in Cameroon have not been studied. The purpose of the current study was to investigate the role of transboundary animal trade in FMDV introduction and spread in the Far North Region of Cameroon through molecular epidemiology of FMDV isolates collected from transboundary and native cattle. Cameroon did not have an FMD control program at the time of this study.

## Materials and Methods

### Study Area

**Figure 1:**
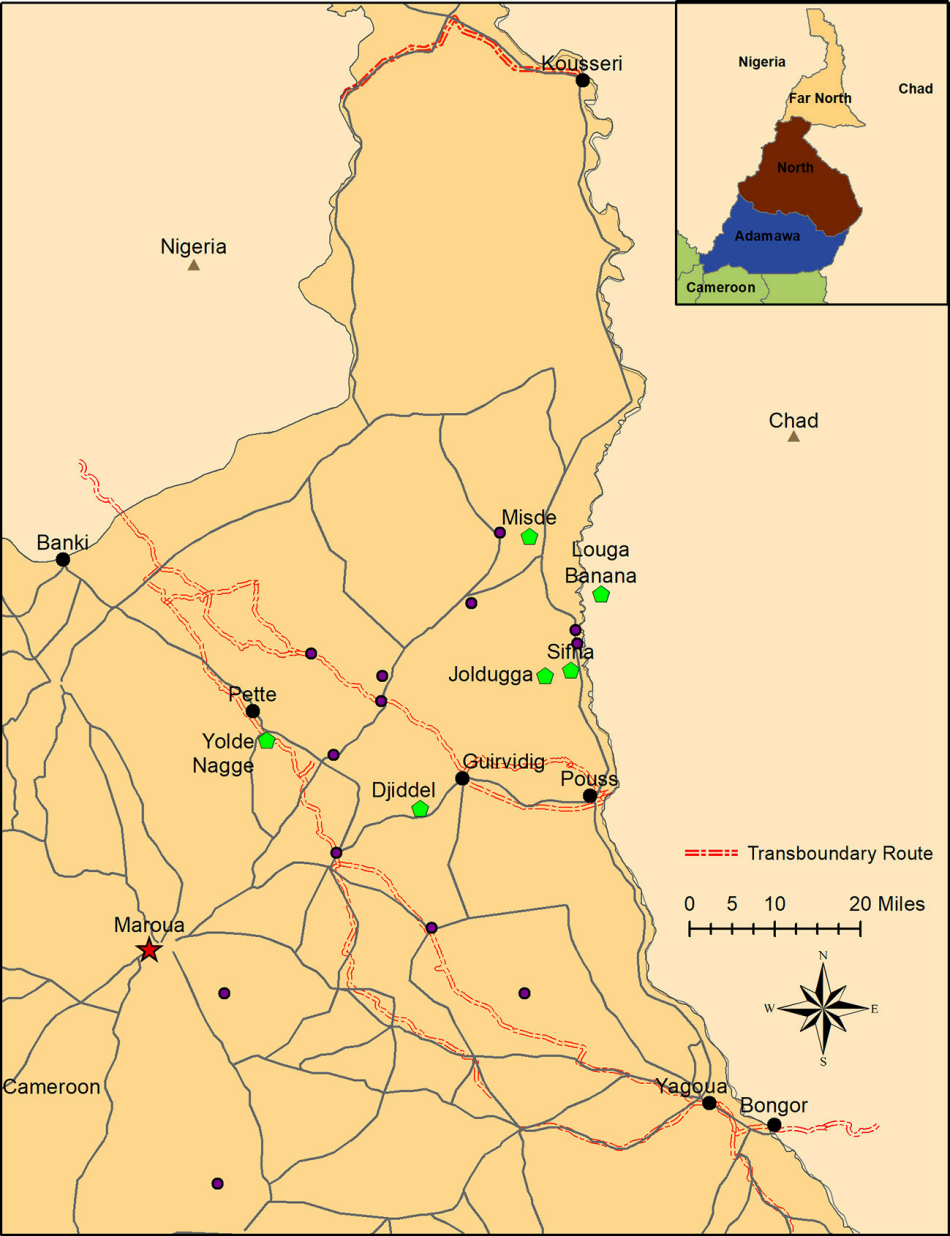
Sampling locations in the Far North Region of Cameroon. Transboundary checkpoints are indicated in black, and locations of native herds with FMD outbreaks are indicated in green. Additionally, FMDV-negative sedentary herds are indicated in blue. Locations of FMDV-negative transhumant herds are not shown because these herds move throughout the region. The usual transhumant routes for these herds are available at MoveBank (https://www.datarepository.movebank.org/handle/10255/move.723). Maroua, the capital of the Far North, is indicated at the star. Inset: location of the Far North Region within Cameroon.

The Far North Region of Cameroon has a total land area of 34,263 km^2^. It is bordered by the North Region to the south, Chad to the east and north, and Nigeria to the west (Figure 1). The climate is semi-arid with a single rainy season. Reduced forage availability during the dry season results in substantial weight loss and increased susceptibility to infectious diseases in cattle (26). Subsistence farming is the primary occupation for most residents. The native livestock population in the Far North Region is primarily cattle raised in a mixture of sedentary (village) and mobile (seasonally transhumant) husbandry systems (19, 27). These two production systems share pastures, water resources, livestock corridors, markets, and veterinary services (28). Additionally, transboundary trade cattle are moved on foot through the Far North Region. The (legal) movement of transboundary trade cattle was exclusively east (Chad and Sudan) to west (Nigeria) during the study period. Native cattle (sedentary and transhumant) were sampled throughout the region and transboundary trade cattle were sampled at the major border control points described below.

### Transboundary Cattle Movement

Transboundary animal trade routes used to move cattle on foot across the Far North Region were mapped by interviewing native cattle herders and transboundary cattle herders accompanying foreign cattle through the Far North. Once general routes were determined, local research staff from CARPA rode the routes on motorcycles and recorded GPS points at 3-s intervals. GPS points of places where transboundary trade cattle stayed overnight and where they watered were also recorded.

Cattle records were collected at the border check points by the Ministry of Livestock, Fisheries and Animal Industries (MINEPIA) veterinary service for tax purposes and during checking of sanitary certificates. The Center for the Support of Research and Pastoralism (CARPA) field team reviewed official government records of cattle passing through border checkpoints each week for which data were available regarding origin and destination, as well as any notes about disease recorded by the Cameroonian veterinary services. These data were summarized to provide context regarding the scale of legal cattle movement and possible origin of viruses.

Summary statistics of numbers of cattle recorded at each transboundary check point along with their reported origin and destination were analyzed in Excel (2013, Microsoft Corp., Redmond, WA, USA).

### Sampling

For the purposes of this study, a herd was defined as the smallest homogenously mixing unit of animals (19). Transboundary trade cattle were sampled at four points along transboundary animal trade routes: Kousseri, Guirvidig, Pétté, and Yagoua (Figure 1). Although two checkpoints were on the same trade route, timing of sample collection ensured that animals were not resampled at different checkpoints. Oropharyngeal fluid (OPF) samples were collected by probang cup (29) two times during each season (dry season: January to April, and wet season: June to September) between 2010 and 2013. Due to logistics in the field, the timing of sampling was not consistent at each location across years. At each visit, a convenience sample of 10 adult cattle were selected by the MINEPIA representative at the checkpoint, with an effort made to collect at least one sample from each distinct herd of trade cattle at the checkpoint that day. Samples collected in 2010–2011 were stored in virus transport media (VTM). In an effort to improve sequence acquisition, samples collected after 2011 were split, and one aliquot was stored in VTM and the other in RNAlater® (Sigma-Aldrich, St. Louis, MO, USA). Additionally, 30 native herds (15 transhumant and 15 sedentary) included in a parallel study (19) were monitored and contacted at least monthly during the study (2010–2014) (Figure 1). Briefly, the transhumant herds were selected randomly from a list of transhumant herds visiting the region between 2008 and 2009, and the sedentary herds were selected in pairs (one with high and one with low contact with transhumant herds) to represent a range of ecological and cultural areas in the Far North [herd definition and selection is detailed in (19)]. If the herder reported clinical signs of FMD in the herd, lesion epithelial tissue or swab samples were collected from affected animals as previously described (30). Samples were also collected from other herds in the area identified by the National Veterinary Laboratory (LANAVET) as having clinical FMD. All samples were kept at 5–10°C during transport (up to 7 days) and then taken to LANAVET in Garoua where they were stored at −80°C until shipping to the Plum Island Animal Disease Center (PIADC), New York, USA. These samples were shipped to PIADC on dry ice by an approved shipper. All samples were collected by CARPA staff following protocols approved by the Ohio State University Institutional Animal Care and Use Committee (protocol #2010A0018) in agreement with the MINEPIA and with permission from the Ministry of Scientific Research and Innovation (MINRESI).

### Virus Isolation

Tissue samples and OPF preserved in VTM were tested by virus isolation (VI). OPF samples were first treated with triclorotrifluoroethane (TTE) to dislodge virus-antibody complexes (31) as previously described (32). Tissue samples were processed by homogenization of 30–50 mg of epithelial tissues in cell culture media (without Fetal Calf Serum) using a bead beater (Qiagen, Valencia, CA) as previously described (32). After processing, 300 μl of each sample was used to infect cells contained in T25 flasks. VI was performed as previously described (33) using an immortalized line of fetal bovine kidney (LFBK) cells expressing the bovine αvβ6 integrin (34).

### FMDV RNA Extraction and rRT-PCR

OPF samples in RNAlater® or in VTM prior to TTE treatment (*N* = 582) and all of the homogenized tissue samples (*N* = 57) were processed for RNA extraction. RNA was extracted using the MagMax-96 viral RNA isolation kit (Ambion, Austin, TX) following the manufacturers protocols on a King Fisher-96 Magnetic Particle Processor (Thermo Scientific, Waltham, MA). Briefly, 50 μl of each sample was added to 150 μl of lysis/binding solution in individual wells of a 96 well plate. After a lysis/binding step, the sample underwent four washes, a drying and a final elution step. RNA was eluted in a final volume of 25 μl of RNAase-free water. The extracted RNA was stored at −70°C until analyzed by real time reverse transcription PCR (rRT-PCR) as previously described (30, 33, 35). Samples were considered positive when Ct values were <40.

### VP1 Sequencing and Phylogenetic Analysis

Fresh viral RNA extracted from rRT-PCR positive samples was used to generate RT-PCR products using SuperScriptIII^TM^ One-Step RT-PCR System with Platinum *Taq* High Fidelity® (Life Technologies, Carlsbad, CA). Universal oligonucleotide primers, designed to amplify the entire P1 region of FMDV were used, as described previously (36). The RT-PCR products were separated in agarose gels, visualized, purified using a QIAquick Gel extraction kit, (Qiagen®, Hilden, Germany), and sequenced using the di-deoxy termination method (BigDye® terminator, Life Technologies) as previously described (19) using additional internal sequencing primers specific to the Cameroon isolates to obtain the complete VP1 coding sequences (Table S1); or by next generation sequencing (NGS) as previously described (37). For sequences obtained using the di-deoxy termination method, chromatograms were viewed using Sequencher® v5.3 (GeneCodes, Ann Arbor, MI) and a consensus sequence was assembled for each isolate's VP1 region. For sequences obtained using the NGS method, VP1 consensus sequences were assembled using CLC Genomics Workbench v11.0 (www.qiagenbioinformatics.com) to map the reads to the closest VP1 reference genome followed by *de novo* assembly of the mapped reads. The minimum depth of coverage for consensus sequence generation was 10 reads/site.

For each serotype, VP1 sequences obtained in this study (**Table 2**) were queried using the BLAST online tool (https://blast.ncbi.nlm.nih.gov), and the first 100 most closely related sequences were aligned along with other publicly available sequences in GenBank from Cameroon and neighboring countries and with previously identified reference sequences for topotype determination (38). Redundant and closely related sequences returned in the BLAST search were removed, and sequences were aligned using MUSCLE (39) implemented in MEGA7 (40). The general time reversible (GTR) model of evolution using a gamma distribution for evolutionary rates (+G) and invariable sites (+I) was identified as the most appropriate model based on the corrected Akaike Information Criterion (AICc). A phylogenetic tree for each serotype was constructed using maximum likelihood, GTR+G+I nucleotide substitution model, and 250 bootstrap replicates implemented in MEGA7. The final consensus trees were visualized in FigTree v.1.4.3 (Rambaut, 2006).

### Statistical Analysis

In order to analyze the patterns of spread of the viruses detected in transboundary trade and native cattle, we examined the relationship between the evolutionary divergence based on complete nucleotide sequences of VP1 (i.e., genetic distance) and the geographic and temporal distance among FMDV isolates within serotypes. The number of base substitutions per site (p-distance) between pairs of sequences was calculated in MEGA7. The geographical distance between sample locations was calculated using GPS coordinates recorded at the time of sample collection. The time difference (in months) was calculated as the time between sampling dates for pairs of samples. Genetic distance was determined at the sequence/sample level, whereas geographic and time distance were determined at the herd level; the correlation analysis was performed at the sample level. A Spearman rank correlation test (41) was performed to analyze the correlation between genetic distance and geographic distance, and between genetic distance and time difference. Using this method, ρ = 1 indicates a perfect monotonic relationship between the genetic distance and either the geographical distance or the time difference, and ρ = 0 indicates no correlation. The asymptotic *t* approximation was used for the calculation of *p*-values. Significance was set at *p* < 0.05, indicating that the value of ρ was significantly different from zero.

## Results

### Cattle Movement Analysis

Observation of transboundary trade routes indicated potential for contact between transboundary trade cattle and native cattle (both sedentary and transhumant) through shared pasture, watering points and markets during transport (Figure 1). Additionally, there were some instances of transboundary trade herds camped overnight with native herds, creating the possibility for direct contact.

Transboundary trade cattle movement data from official government records of cattle herds passing through border check points were available for 3,273 herds for the period January 2008 to December 2013, with varying consistency for each of four check points: Kousseri, Guirvidig, Pétté, and Yagoua (Figure 1). All reported origins of transboundary trade herds were in Chad, and all reported destinations were in Nigeria.

**Figure 2:**
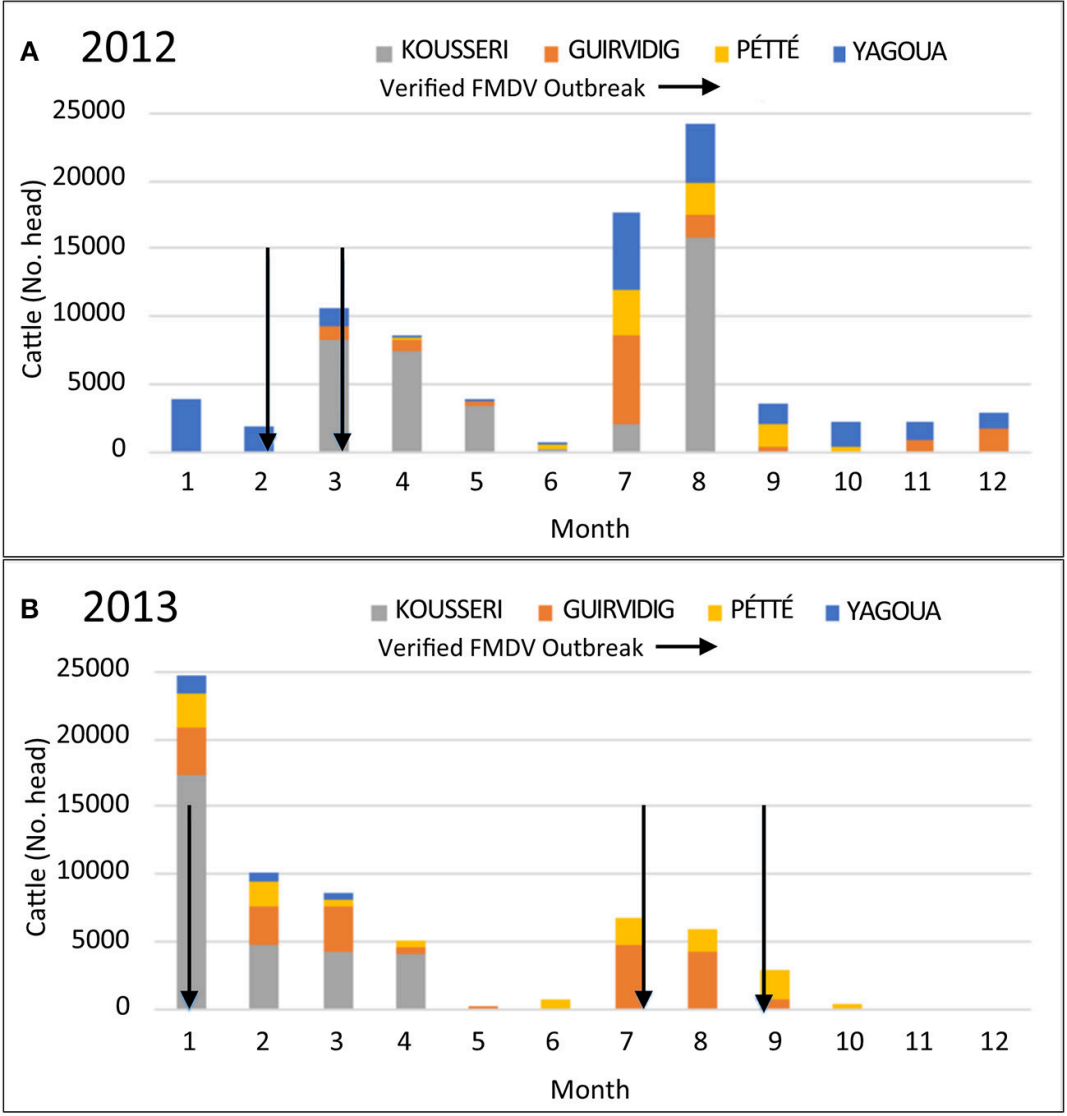
Numbers of cattle passing through 4 different border checkpoints in the Far North, Cameroon by month for **(A)** 2012 and **(B)** 2013. Verified FMD outbreaks are indicated with arrows. FMD outbreaks were verified by viral genomic sequencing of RNA from vesicle epithelium samples collected from clinically ill animals.

The years 2012 and 2013 had the most consistent data recording and showed variable geographic and seasonal patterns in transboundary movements (Figure 2). In general, transboundary movement decreased during the months with the highest precipitation levels (May and June) as well as in October and November, which is typically a time of transhumance movements for native herders in the Far North. Dates of genetically confirmed FMD outbreaks (arrows, Figure 2) generally coincided with transboundary movement, however there was not a clear or consistent pattern.

### rRT-PCR, Virus Isolation, and VP1 Sequencing

Of 355 OPF samples from transboundary trade cattle preserved in VTM, 24 (6.8%) were positive for FMDV RNA by rRT-PCR but only 2 of these yielded infectious virus. Additionally, 18/227 (7.9%) OPF samples preserved in RNAlater® were positive by rRT-PCR (Table 1). Sample preservation in RNAlater® does not allow for virus isolation. There was no significant difference in detection of FMDV RNA in samples preserved in RNAlater® compared to VTM (χ^2^ = 0.13, *df* = 1, *p* = 0.71). In total, 42 out of 582 samples (7.2%) tested positive by rRT-PCR. VP1 coding sequences were obtained from 6 OPF samples, of which 4 were serotype O and 2 were SAT2 (Table 2).

**Table 1:** FMDV virus isolation and rRT-PCR results from the transboundary (subclinical) and native (clinical) cattle samples.

**Cattle group**	**Sample type**	**Samples tested**	**Virus isolation Positive (%)**	**rRT-PCR Positive (%)**	Sequences obtained^*^
Transboundary	Probang in VTM	355	2 (0.6%)	24 (6.8%)	5
Transboundary	Probang in RNAlater®	227	NA	18 (7.9%)	1
Native	Vesicle epithelium in VTM	57	17 (29.8%)	40 (70.2%)	19

* *Sequences were obtained from all 19 virus isolates and 6 additional unpassaged samples*.

**Table 2:** Sample information and accession numbers for VP1 sequences obtained in this study.

	**Sample ID**	**Sequence name**	**Sample type**	**Collection date**	**Collection location**	**rtRT-PCR CT values**	**VI**	**FMDV serotype**	**Genbank accession number**
Transboundary trade cattle	G3836	O/CAR/G3836/2012	Probang in VTM	20-Mar-12	Pouss	38.27	Pos	O	KY581682
	G4765	O/CAR/G4765/2013	Probang in VTM	30-Jul-13	Pétté	>40.00	Pos	O	KY581678
	G4848	O/CAR/G4848/2013	Probang in VTM	6-Sep-13	Kousseri	34.45	Neg	O	KY581677
	G4849	O/CAR/G4849/2013	Probang in VTM	6-Sep-13	Kousseri	35.23	Neg	O	KY581676
	G3808	SAT2/CAR/G3808/2012	Probang in VTM	6-Mar-12	Kousseri	35.6	Neg	SAT 2	KY581673
	B01070	SAT2/CAR/B001070/2012	Probang in RNA Later®	6-Mar-12	Kousseri	27.65	NA	SAT 2	KY581675
Native (sedentary and transhumant) cattle	LAN4	A/CAR/LAN4/2014	Vesicle epithelium in VTM	30-Jan-14	Sifna	13.09	Pos	A	MH383069
	LAN9	A/CAR/LAN9/2014	Vesicle epithelium in VTM	30-Jan-14	Sifna	15.10	Pos	A	MH383070
	LAN10	A/CAR/LAN10/2014	Vesicle epithelium in VTM	30-Jan-14	Sifna	15.05	Pos	A	MH383071
	LAN11	A/CAR/LAN11/2014	Vesicle epithelium in VTM	30-Jan-14	Sifna	15.74	Pos	A	MH383072
	LAN12	A/CAR/LAN12/2014	Vesicle epithelium in VTM	30-Jan-14	Sifna	19.23	Pos	A	MH383073
	LAN14	A/CAR/14/2014	Vesicle epithelium in VTM	30-Jan-14	Sifna	15.16	Pos	A	KY581683
	LAN15	A/CAR/LAN15/2014	Vesicle epithelium in VTM	30-Jan-14	Sifna	14.83	Pos	A	MH383074
	LAN16	A/CAR/LAN16/2014	Vesicle epithelium in VTM	30-Jan-14	Sifna	17.88	Pos	A	MH383075
	LAN17	A/CAR/LAN17/2014	Vesicle epithelium in VTM	30-Jan-14	Sifna	22.13	Pos	A	MH383076
	G3856	O/CAR/G3856/2012	Vesicle epithelium in VTM	27-Mar-12	Yolde Nagge	15.07	Pos	O	KY581681
	G3857	O/CAR/G3857/2012	Vesicle epithelium in VTM	27-Mar-12	Yolde Nagge	13.95	Pos	O	MH383077
	G4250	O/CAR/G4250/2013	Vesicle epithelium in VTM	10-Jan-13	Louga Banana	34.81	Pos	O	MH383078
	G4258	O/CAR/G4258/2013	Vesicle epithelium in VTM	10-Jan-13	Louga Banana	18.64	Pos	O	KY581680
	G4260	O/CAR/G4260/2013	Vesicle epithelium in VTM	10-Jan-13	Lugga Banana	31.94	Pos	O	KY581679
	G4262	O/CAR/G4262/2013	Vesicle epithelium in VTM	10-Jan-13	Lugga Banana	16.99	Neg	O	MH383079
	G4268	O/CAR/G4268/2013	Vesicle epithelium in VTM	10-Jan-13	Joldugga	23.96	Neg	O	MH383080
	G3796	SAT2/CAR/G3796/2012	Vesicle epithelium in VTM	26-Feb-12	Misde	14.76	Pos	SAT2	KY581674
	G3852	SAT2/CAR/G3852/2012	Vesicle epithelium in VTM	26-Mar-12	Djiddel	15.48	Pos	SAT2	KY581672
	G3853	SAT2/CAR/G3853/2012	Vesicle epithelium in VTM	26-Mar-12	Djiddel	15.13	Pos	SAT2	KY581671

In total, 57 tissue samples were collected from 8 outbreaks in native cattle. Overall, 40/57 (70.2%) samples, representing all 8 outbreaks, were positive by rRT-PCR, and 17/57 (29.8%) samples, representing 6 outbreaks, were positive by virus isolation (Table 1). VP1 coding sequences were obtained from all 17 virus isolates plus an additional two raw (unpassaged) samples (total 19 samples from 6 outbreaks), of which 7 were serotype O, 3 were SAT2, and 9 were serotype A.

### Phylogenetic Analysis

**Figure 3:**
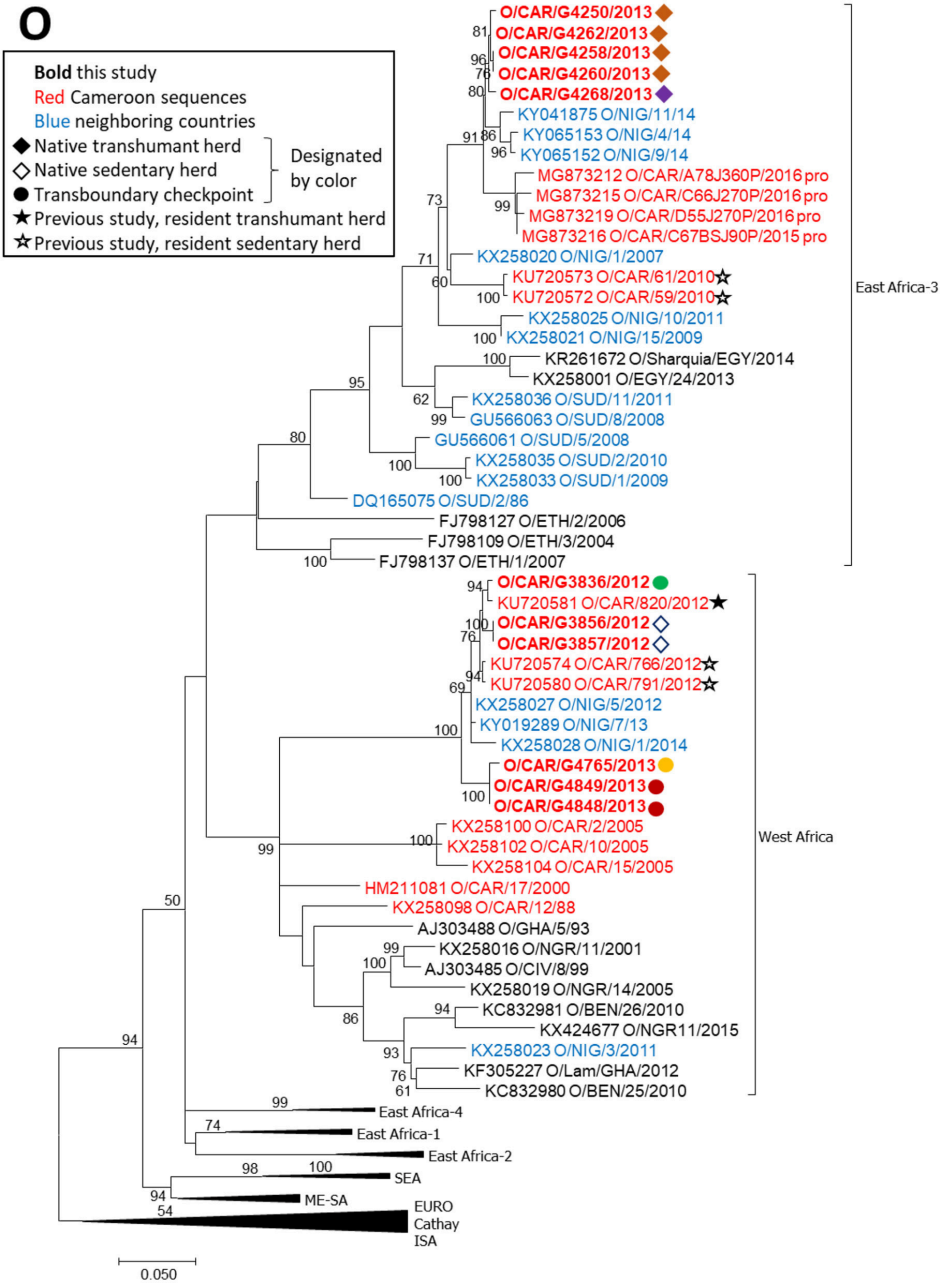
VP1 phylogenetic tree for serotype O inferred using the maximum likelihood method based on the GTR+G+I model. Branch lengths indicate the number of substitutions per site. Bootstrap values >50% are indicated at the nodes. The type of herd (transboundary, native sedentary, native transhumant) from which the samples were collected is indicated by the symbol, and unique herds are indicated by the color of the symbol.

FMDV serotype O VP1 sequences obtained in this study belonged to two topotypes, East Africa-3 and West Africa (Figure 3). Five sequences, collected from 2 native mobile herds in 2013, were within topotype East Africa-3 and grouped with sequences collected from Nigeria in 2014 and from the Adamawa region of Cameroon in 2016. The topotype O/East Africa-3 sequences obtained in this study were also closely related to sequences from native sedentary herds in 2010 (19) (*p*-distance 0.058–0.063). Six sequences, collected from one native sedentary herd and from asymptomatic animals at three transboundary checkpoints, were within topotype O/West Africa and were closely related to sequences collected in Nigeria in 2012–2014 as well as sequences obtained from native sedentary and mobile herds in a previous study in Cameroon in 2012 (*p*-distance 0.011–0.029) (19).

**Figure 4:**
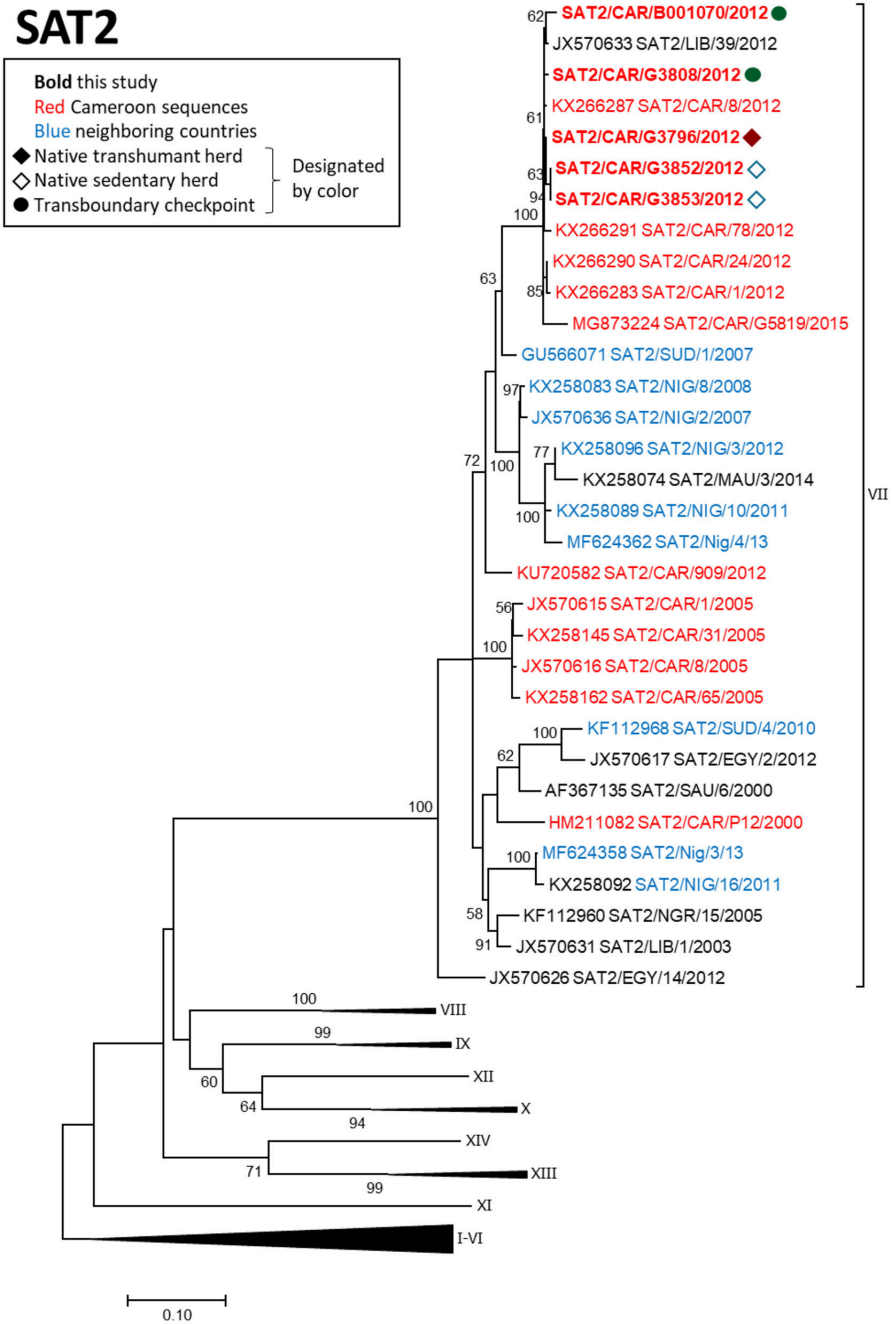
VP1 phylogenetic tree for serotype SAT2 inferred using the maximum likelihood method based on the GTR+G+I model. Branch lengths indicate the number of substitutions per site. Bootstrap values >50% are indicated at the nodes. The type of herd (transboundary, native sedentary, native transhumant) from which the samples were collected is indicated by the symbol, and unique herds are indicated by the color of the symbol.

FMDV serotype SAT2 VP1 sequences obtained in this study were collected in 2012 from one native sedentary herd (2 sequences), one native mobile herd (1 sequence), and one transboundary checkpoint (2 sequences) (Figure 4). The 5 sequences were all collected within a 1-month period during 2012 and grouped within topotype SAT2/VII with viruses collected in the Adamawa region of Cameroon and in Libya during 2012 (*p*-distance 0.003–0.091). Other regional isolates collected in previous years were more distantly related.

**Figure 5:**
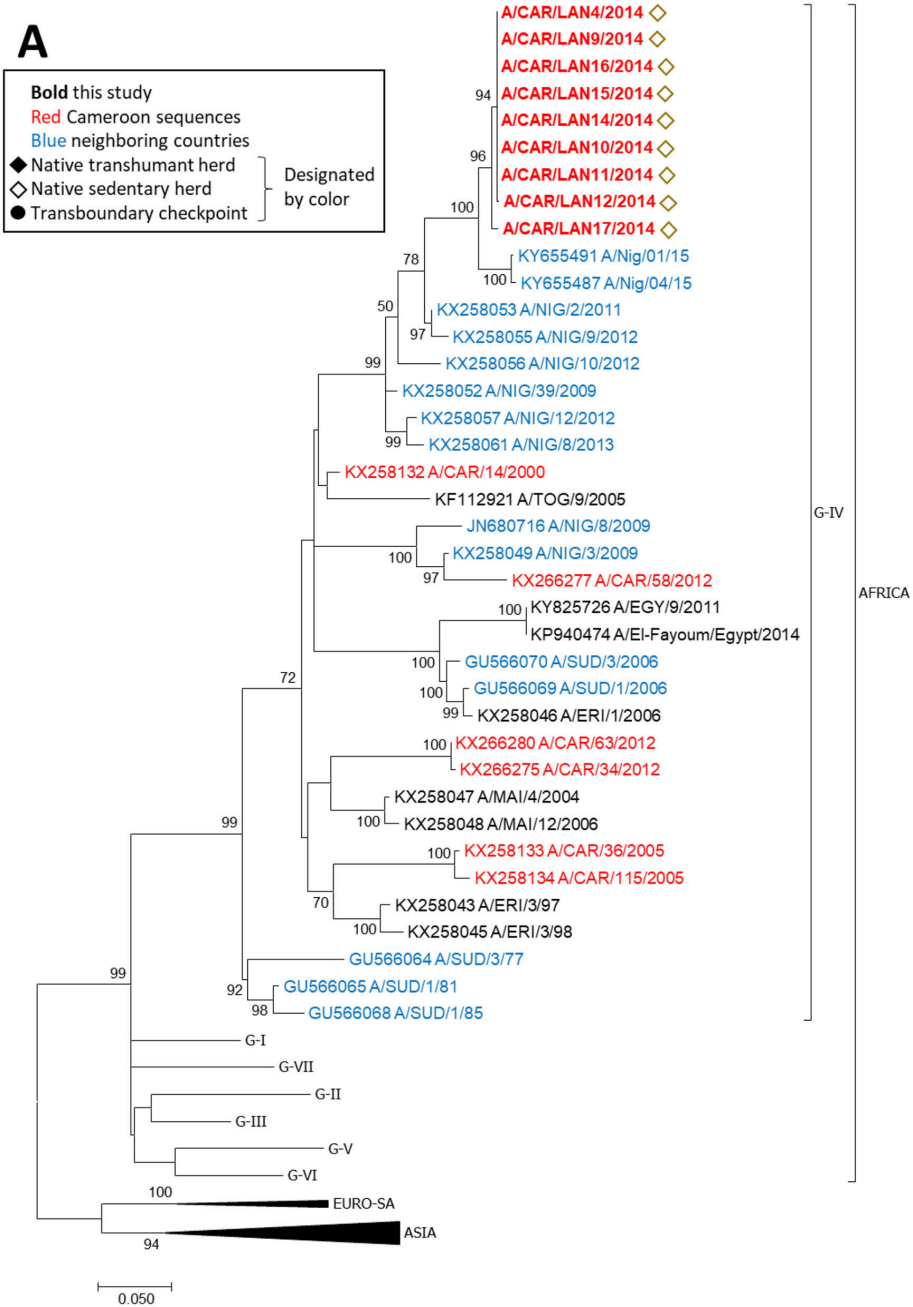
VP1 phylogenetic tree for serotype A inferred using the maximum likelihood method based on the GTR+G+I model. Branch lengths indicate the number of substitutions per site. Bootstrap values >50% are indicated at the nodes. The type of herd (transboundary, native sedentary, native transhumant) from which the samples were collected is indicated by the symbol, and unique herds are indicated by the color of the symbol.

FMDV serotype A sequences obtained in this study were collected in 2014 from a single outbreak in a native herd (Figure 5). The nine sequences were nearly identical (maximum 6 nucleotides different) and clustered within topotype A/AFRICA/G-IV with isolates collected in Nigeria in 2009–2015 (*p*-distance 0.034–0.085). The isolates collected in the current study were more distantly related to viruses collected in the North West (*p*-distance 0.135–0.141) and Adamawa (*p*-distance 0.137–0.143) regions of Cameroon in 2012 and 2005, respectively.

### Correlation Analysis

All serotype A sequences were obtained at the same location and time, and serotype O topotype East Africa-3 sequences were obtained at the same time from only 2 locations, therefore correlation analysis was not performed for these serotypes. We performed correlation analysis for serotype O topotype West Africa sequences (*n* = 6) and for serotype SAT2 sequences (*n* = 5). Strains within each of the serotypes were closely related, with genetic distances (p-distance) ranging from 0 to 0.03 for serotype O, and 0 to 0.02 for SAT2 (Table 3). Geographic distances ranged from 0 to 137 km for serotype O, and 0 to 140 km for SAT2. Time between samples ranged from 0 to 17.9 months for serotype O, and 0 to 1 month for SAT2. When the two serotypes were analyzed separately, a significant positive correlation between genetic distance and time was observed for serotype O (ρ = 0.71, *p* = 0.003), and the positive correlation between genetic distance and geographical distance for serotype SAT2 approached significance (ρ = 0.54, *p* = 0.1).

**Table 3:** Genetic, geographic, and temporal distance between pairs of sequences from transboundary and native (in bold) cattle.

**FMDV Serotype**	**Sequence pair**		**Genetic distance**	**Geographical distance (km)**	**Time Dif. (months)**
FMDV serotype O	ID G4848	ID G4849	0.00	0	0
	**ID G3856**	**ID G3857**	0.00	0	0
	ID G3836	**ID G3856**	0.01	60	0.3
	ID G3836	**ID G3857**	0.01	60	0.3
	ID G4848	ID G4765	0.01	135	1.6
	ID G4849	ID G4765	0.01	135	1.6
	ID G3836	ID G4848	0.02	134	17.9
	ID G3836	ID G4849	0.02	134	17.9
	ID G3836	ID G4765	0.03	0	16.3
	**ID G3856**	ID G4765	0.03	60	16
	**ID G3857**	ID G4765	0.03	60	16
	**ID G3856**	ID G4848	0.03	137	17.6
	**ID G3856**	ID G4849	0.03	137	17.6
	**ID G3857**	ID G4848	0.03	137	17.6
	**ID G3857**	ID G4849	0.03	137	17.6
FMDV serotype SAT2	**ID G3852**	**ID G3853**	0	0	0
	**ID G3796**	**ID G3852**	0.01	55	1
	**ID G3796**	**ID G3853**	0.01	55	1
	**ID G3796**	ID G3808	0.01	86	0.3
	ID G3808	**ID G3852**	0.01	140	0.7
	ID G3808	**ID G3853**	0.01	140	0.7
	ID B01070	ID G3808	0.02	0	0
	ID B01070	**ID G3796**	0.02	86	0.3
	ID B01070	**ID G3852**	0.02	140	0.7
	ID B01070	**ID G3853**	0.02	140	0.7

## Discussion

Foot and mouth disease is endemic in Cameroon, where most livestock are managed in subsistence and transhumance husbandry systems with animals often moved on foot within the country and between neighboring countries. Transboundary movements of animals are suggested to contribute to outbreaks in endemic regions (15, 42, 43), and FMDV spread in Africa has been associated with animal movement either via pastoralism or animal trade (9, 14, 44). The current study investigated the molecular epidemiology of FMDV in the Far North Region of Cameroon between 2010 and 2014 based on samples collected from mobile and sedentary native cattle and transboundary trade cattle monitored through movement at official border check points.

In the current study, the documented, legal transboundary movement of cattle was exclusively east (Chad) to west (Nigeria), with no consistent pattern of monthly peak animal movement in 2012-2013, although the fewest animals were consistently recorded in May-June and October-December. The lulls in transboundary traffic corresponded to periods of long-distance travel for native transhumant herds (45), suggesting a relative scarcity of forage in the region, which may contribute to fewer transboundary animals moving through the region at those times. Outbreaks tended to occur during periods of transboundary trade movement but the pattern was not consistent. For example, outbreaks coincided with increased transboundary movement during March 2012, January 2013, and July 2013, however outbreaks were not recorded during times of increased transboundary movement in July and August 2012. Transboundary traffic is expected to result in contact between transboundary and native cattle, facilitating disease spread. The current study analyzed outbreaks verified by genetic sequencing and official government records of transboundary movement of trade cattle. However, previous reports have indicated only about 10% of transboundary trade passes through official channels in some parts of Africa (9). Unrecorded animal movement, along with limited outbreak reporting and sampling in the region, likely contributed to the inconsistent association of outbreaks with higher numbers of official transboundary animal movements in the current study.

The results of the phylogenetic analyses are consistent with previous reports for Cameroon regarding serotype distribution and genetic lineage relationships (17–19, 46), and are also consistent with reports for other countries in the region (21–23, 47). Overall, the current study indicated that viruses from Cameroon share commonalities with viruses from West, East, and North Africa, suggesting regional transmission between Cameroon and neighboring countries, although the direction of transmission is unclear.

The rate of evolution for a gene (i.e., the molecular clock) can be used to estimate the date of the most recent common ancestor of a pair of sequences in a phylogeny, however molecular clock estimates are inaccurate for small genetic distances (48, 49). For example, the average rate of evolution of the VP1 coding region has been estimated at 5.95 × 10^−3^ substitutions/site/year for serotype O, and 1.19 × 10^−2^ substitutions/site/year for serotype A (50), and the time to the most recent common ancestor between a pair of sequences is usually measured in years. Consequently, patterns of transmission can be difficult to discern at the small genetic distances and time between sequences obtained in the current study using phylogenetic analysis. To supplement the phylogenetic analysis, the current study used correlation analysis to help discern patterns of FMDV transmission. A positive correlation between genetic distance and time suggests a widespread contemporaneous epidemic in which mutation occurs simultaneously in different locations, as has been reported for regional transmission of Ebola virus (51). Conversely, a positive correlation between genetic distance and geographic distance suggests a wave of a virus epidemic moving through a region with little transmission behind the leading edge, as has been reported for rabies virus (52).

FMDV serotype O viruses obtained in this study grouped into two topotypes, East Africa-3 and West Africa. Within each topotype, viruses from this study grouped with viruses collected in Cameroon in recent studies (19, 37) and with other regional viruses from neighboring countries, suggesting continuous regional transmission of both topotypes. Although one transboundary trade virus (G3836) was collected prior to an outbreak in a native herd, an ancestral relationship between these transboundary and native viruses could not be established because they were too closely related. The strong positive correlation (ρ = 0.71) between genetic distance and time for serotype O topotype West Africa viruses (*n* = 6) in this study further supports the hypothesis that type O viruses transmit and evolve continually in the region. Additionally, previous work in the Far North Region of Cameroon indicated continuous circulation of serotype O in that region, with sustained chains of transmission (20).

FMDV serotype SAT2 viruses isolated from asymptomatic transboundary trade cattle in 2012 grouped closely with viruses isolated in clinical samples from native cattle and with viruses from North Africa (Libya) isolated in the same year, likely reflecting a region-wide epidemic caused by SAT2 in 2012 (44, 53). Although sample collection from transboundary trade cattle occurred prior to sample collection from the native sedentary animals, directionality of transmission could not be determined from our data due to the high similarity among the viruses, as was the case for serotype O. Although not statistically significant, the positive correlation (ρ = 0.54) observed between genetic distance and geographic distance for SAT2 isolates (*n* = 5) further supports a quick-moving outbreak advancing over a large geographic area with little transmission behind the leading edge of the outbreak. Additionally, a previous study in the Far North Region of Cameroon indicated that SAT2 outbreaks may be caused by repeated introductions of the virus (20), which is consistent with the more phylogenetically distant relationships between the SAT2 viruses obtained in the current study and those collected in previous years in Cameroon and Nigeria. A previous study from the Adamawa region of Cameroon also suggested that movement of cattle was responsible for the introduction of FMDV SAT2 from East Africa (18).

In this study, serotype A viruses were represented by a single outbreak from a native sedentary herd in 2014, and the viruses grouped with viruses from Nigeria collected between 2009 and 2015, indicating a pattern of regional transmission similar to that observed for serotype O. Previous studies have also indicated a close relationship between Nigerian FMDV serotype A isolates and FMDV isolates from Cameroon (21), most likely due to animal trade (54). In contrast, isolates collected in other regions of Cameroon in 2012 and 2005 were more distantly related to the sequences obtained in the current study, which suggests a pattern of repeated introductions. A previous study suggested that serotype A in Cameroon has an epidemic pattern of distribution (similar to serotype SAT2) with short periods of sustained transmission (20), which is consistent with the phylogenetic relationships among serotype A viruses reported in the current study. Additionally, a previous study showed a close relationship between serotype A viruses collected in Sudan and viruses collected elsewhere in the region (23), suggesting long-distance animal movement is important for spread of this serotype. Transboundary trade may introduce new serotype A strains into Cameroon, and may facilitate spread of this serotype within Cameroon and into Nigeria. However, no serotype A viruses were obtained from transboundary animals in the current study, and the origin or direction of movement of the viruses cannot be determined with the limited data available.

Transboundary and native cattle shared closely related viruses in the current study, indicating transmission occurs among these groups of cattle, although infection from some other common source is possible. However, the analysis is limited due to the paucity of publicly available FMDV sequences from the region. Samples from Cameroon, Sudan, and Nigeria are limited to a handful of sampling periods, and there is a complete lack of samples from Chad. Consequently, the phylogenetic analysis suggests regional patterns of transmission, however the directionality of transmission cannot be determined.

Additionally, the proportion of samples that yielded either infectious virus or sequence data in this study was small relative to the number of samples collected. This reflects the challenge of collecting field samples in distant areas with limited access to adequate sample preservation facilities. Due to sampling logistics and limitations in the field, samples were stored on ice packs (5–10°C) for up to 7 days prior to transfer to the laboratory and storage at −80°C. We attempted to increase FMDV RNA detection through sample preservation in RNA stabilizing solution (RNAlater®), however this did not significantly increase detection, and preservation in RNAlater® prevented isolation of live virus from these samples. In the current study, FMDV RNA was detected in 6.8% (*n* = 355) of OPF samples preserved in VTM, while infectious virus was isolated from only 0.6% of these samples. In comparison, a previous study in Cameroon isolated infectious virus from 3.5% of OPF samples (18). The limited FMDV RNA detection and infectious virus isolation in the current study might be due to sample degradation. The limited number of sequences obtained may also be due to sample degradation or to failure of the primers used to universally amplify the P1 region of some of the FMDV variants. We used two approaches to maximize the number of sequences obtained; by testing multiple primers tailored to amplify regional viruses for Sanger sequencing and by next generation sequencing using the Nextera XT DNA library platform.

The current study describes the molecular epidemiology of FMDV isolates collected from transboundary trade and native cattle in the Far North Region of Cameroon between 2010 and 2014. The evidence showed that FMDV serotypes O, SAT2, and A were present in the Far North Region of Cameroon, and transboundary trade cattle were likely involved in the epidemiology of FMDV in this region. The close genetic relationships between viruses from transboundary trade cattle and viruses from native cattle suggests transmission between these groups. Phylogenetic analysis indicated two topotypes of FMDV serotype O circulating continuously in Cameroon. In contrast, serotype SAT2 viruses were likely introduced more recently, and serotype A viruses may be introduced repeatedly and circulate for a period after introduction. The current study adds to the understanding of FMDV epidemiology in Cameroon and the region. However, this study is limited by the scarcity of FMDV sequences from Central Africa. Further sampling in the region is necessary to investigate patterns of transmission and the role of transboundary trade cattle in FMDV epidemiology. Better understanding of viral epidemiology will allow targeted control strategies (e.g., temporary animal movement restrictions and/or vaccination). Our results provide context for FMDV transmission in Cameroon and the larger Sahel region of Africa. This information is critical to prioritize resources within Cameroon's recently initiated FMDV vaccination campaigns.

## Data Availability

The datasets analyzed during the current study are available from the authors upon reasonable request.

## Author Contributions

MB and CB performed the analyses and drafted the manuscript. RG and LR conceived the study, participated in the design and coordination, and helped to draft the manuscript. SD and SA participated in the design and coordination of the study, and contributed to sample acquisition in Cameroon. MM oversaw data acquisition and analysis. SP oversaw and executed the sequence acquisition studies. JA participated in the design and coordination, and helped to draft the manuscript. All authors read and approved of the final manuscript.

### Conflict of Interest Statement

The authors declare that the research was conducted in the absence of any commercial or financial relationships that could be construed as a potential conflict of interest.

## Supplementary Material

Table S1 Internal sequencing primers used to obtain the complete Cameroonian VP1 sequences.

## References

[1] Knight-Jones TJD Rushton J. The economic impacts of foot and mouth disease – what are they, how big are they and where do they occur? Prev Vet Med. (2013) 112:161–73. 10.1016/j.prevetmed.2013.07.013 23958457 PMC3989032

[2] Arzt J Juleff N Zhang Z Rodriguez LL. The pathogenesis of foot-and-mouth disease I: viral pathways in cattle. Transbound Emerg Dis. (2011) 58:291–304. 10.1111/j.1865-1682.2011.01204.x 21366894

[3] Arzt J Baxt B Grubman MJ Jackson T Juleff N Rhyan J. The pathogenesis of foot-and-mouth disease II: viral pathways in swine, small ruminants, and wildlife; myotropism, chronic syndromes, and molecular virus-host interactions. Transbound Emerg Dis. (2011) 58:305–26. 10.1111/j.1865-1682.2011.01236.x 21672184

[4] Knight-Jones TJD McLaws M Rushton J. Foot-and-mouth disease impact on smallholders - what do we know, what don't we know and how can we find out more? Transbound Emerg Dis. (2016) 64:1079–94. 10.1111/tbed.12507 27167976 PMC5516236

[5] Bachrach HL. Foot-and-mouth disease. Annu Rev Mircrobiol. (1968) 22:201–44. 4301615 10.1146/annurev.mi.22.100168.001221

[6] Pereira HG. Subtyping of foot-and-mouth disease virus. In: Mackowiak C Regamey RH, editors. Developments in Biological Standardization. Basel: Karger (1977). p. 333–63. 198283

[7] Knowles NJ Samuel AR. Molecular epidemiology of foot-and-mouth disease virus. Virus Res. (2003) 91:65–80. 10.1016/S0168-1702(02)00260-5 12527438

[8] Rweyemamu M Roeder P Mackay D Sumption K Brownlie J Leforban Y. Epidemiological patterns of foot-and-mouth disease worldwide. Transbound Emerg Dis. (2008) 55:57–72. 10.1111/j.1865-1682.2007.01013.x 18397509

[9] Di Nardo A Knowles NJ Paton DJ. Combining livestock trade patterns with phylogenetics to help understand the spread of foot and mouth disease in sub-Saharan Africa, the Middle East, and Southeast Asia. Rev Sci Tech (2011) 30:63–85. 21809754 10.20506/rst.30.1.2022

[10] Samuel AR Knowles NJ. Foot-and-mouth disease type O viruses exhibit genetically and geographically distinct evolutionary lineages (topotypes). J Gen Virol. (2001) 82:609–21. 10.1099/0022-1317-82-3-609 11172103

[11] Sutmoller P Barteling SS Olascoaga RC Sumption KJ. Control and eradication of foot-and-mouth disease. Virus Res. (2003) 91:101–44. 10.1016/S0168-1702(02)00262-9 12527440

[12] Green DM Kiss IZ Kao RR. Modelling the initial spread of foot-and-mouth disease through animal movements. Proc R Soc B Biol Sci. (2006) 273:2729–35. 10.1098/rspb.2006.3648 17015320 PMC1635508

[13] Mohapatra JK Subramaniam S Pandey LK Pawar SS De A Das B. Phylogenetic structure of serotype A foot-and-mouth disease virus: global diversity and the Indian perspective. J Gen Virol. (2011) 92:873–9. 10.1099/vir.0.028555-0 21228130

[14] Sangare O Bastos ADS Venter EH Vosloo W. A first molecular epidemiological study of SAT-2 type foot-and-mouth disease viruses in West Africa. Epidemiol Infect. (2004) 132:525–32. 10.1017/S0950268803001833 15188721 PMC2870131

[15] Subramaniam S Pattnaik B Sanyal A Mohapatra JK Pawar SS Sharma GK. Status of foot-and-mouth disease in India. Transbound Emerg Dis. (2013) 60:197–203. 10.1111/j.1865-1682.2012.01332.x 22551096

[16] Ekue NF Tanya VN Ndi C. Foot-and-mouth disease in Cameroon. Trop Anim Health Prod. (1990) 22:34–6. 2157306 10.1007/BF02243496

[17] Ferris NP Donaldson AI. The World Reference Laboratory for Foot and Mouth Disease: a review of thirty-three years of activity (1958-1991). Rev Sci Tech. (1992) 11:657–84. 1335302 10.20506/rst.11.3.626

[18] Bronsvoort BMdC Radford AD Tanya VN Nfon C Kitching RP Morgan KL. Molecular epidemiology of foot-and-mouth disease viruses in the Adamawa Province of Cameroon. J Clin Microbiol. (2004) 42:2186–96. 10.1128/jcm.42.5.2186-2196.2004 15131187 PMC404612

[19] Ludi A Ahmed Z Pomeroy LW Pauszek SJ Smoliga GR Moritz M. Serotype diversity of foot-and-mouth-disease virus in livestock without history of vaccination in the Far North Region of Cameroon. Transbound Emerg Dis. (2016) 63:e27–38. 10.1111/tbed.12227 24735162 PMC4499489

[20] Pomeroy LW Bjornstad ON Kim H Jumbo SD Abdoulkadiri S Garabed R. Serotype-specific transmission and waning immunity of endemic foot-and-mouth disease virus in Cameroon. PLoS ONE (2015) 10:e0136642. 10.1371/journal.pone.0136642 26327324 PMC4556668

[21] Ehizibolo DO Perez AM Carrillo C Pauszek S AlKhamis M Ajogi I. Epidemiological analysis, serological prevalence and genotypic analysis of foot-and-mouth disease in Nigeria 2008-2009. Transbound Emerg Dis. (2014) 61:500–10. 10.1111/tbed.12054 23347819

[22] Sahle M Dwarka RM Venter EH Vosloo W. Comparison of SAT-1 foot-and-mouth disease virus isolates obtained from East Africa between 1971 and 2000 with viruses from the rest of sub-Saharan Africa. Arch Virol. (2007) 152:797–804. 10.1007/s00705-006-0893-x 17187294

[23] Habiela M Ferris NP Hutchings GH Wadsworth J Reid SM Madi M. Molecular characterization of foot-and-mouth disease viruses collected from Sudan. Transbound Emerg Dis. (2010) 57:305–14. 10.1111/j.1865-1682.2010.01151.x 20626708

[24] FAO Panorama report on food and agriculture statistics, GCP/GLO/208/BMG Country STAT for Sub-Saharan Africa Rome, Italy (2009). Available online at: http://www.abhatoo.net.ma/maalama-textuelle/developpement-durable/economie-durable/agriculture/agro-industrie/conservation-des-aliments/rapport-panorama-i-sur-les-statistiques-agricoles-et-alimentaires-cameroun (Accessed Aug 28, 2017).

[25] Seignobos C Iyébi-Mandjek O editors. Atlas de la Province Extrême-Nord Cameroun. Paris: Éditions de l'IRD/MINREST/INC (2000).

[26] Healy Profitos JM Moritz M Garabed R What to do with chronically sick animals? Pastoralists' management strategies in the far north region of Cameroon. Pastoral Res PolicyPract. (2013) 3:8 10.1186/2041-7136-3-8 PMC4193801 25309717

[27] Moritz M Crop–livestock interactions in agricultural and pastoral systems in West Africa. Agric Hum Values (2010) 27:119–28. 10.1007/s10460-009-9203-z

[28] Njoya A Bouchel D Ngo Tama AC Moussa C Martrenchar A Letenneur L Systèmes d'élevage et productivité des bovins en milieu paysan. World Anim Rev. (1997) 89:12–23.

[29] Sutmoller P Gaggero C Foot-and-mouth disease carriers. Vet Rec. (1965) 77:968–9. 10.1136/vr.77.33.968 5890082

[30] Arzt J Pacheco JM Rodriguez LL. The early pathogenesis of foot-and-mouth disease in cattle after aerosol inoculation. Identification of the nasopharynx as the primary site of infection. Vet Pathol. (2010) 47:1048–63. 10.1177/0300985810372509. 20587691

[31] Sutmoller P Cottral GE. Improved techniques for the detection of foot-and-mouth disease virus in carrier cattle. Arch Gesamte Virusforschung (1967) 21:170–7. 4298422 10.1007/BF01241441

[32] Pacheco JM Smoliga GR O'Donnell V Brito BP Stenfeldt C Rodriguez LL. Persistent foot-and-mouth disease virus infection in the nasopharynx of cattle; tissue-specific distribution and local cytokine expression. PLoS ONE (2015) 10:e0125698. 10.1371/journal.pone.0125698 25996935 PMC4440813

[33] Pacheco JM Arzt J Rodriguez LL. Early events in the pathogenesis of foot-and-mouth disease in cattle after controlled aerosol exposure. Vet J. (2010) 183:46–53. 10.1016/j.tvjl.2008.08.023 18930417

[34] LaRocco M Krug PW Kramer E Ahmed Z Pacheco JM Duque H. A continuous bovine kidney cell line constitutively expressing bovine αVβ6 integrin has increased susceptibility to foot-and-mouth disease virus. J Clin Microbiol. (2013) 51:1714–20. 10.1128/JCM.03370-12 23515553 PMC3716081

[35] Callahan JD Brown F Osorio FA Sur JH Kramer E Long GW. Use of a portable real-time reverse transcriptase polymerase chain reaction assay for rapid detection of foot-and-mouth disease virus. J AmVet Med Assoc. (2002) 220:1636–42. 10.2460/javma.2002.220.1636 12051502

[36] Xu L Hurtle W Rowland JM Casteran KA Bucko SM Grau FR. Development of a universal RT-PCR for amplifying and sequencing the leader and capsid-coding region of foot-and-mouth disease virus. J Virol Methods (2013) 189:70–6. 10.1016/j.jviromet.2013.01.009 23380590

[37] Bertram MR Delgado A Pauszek SJ Smoliga GR Brito B Stenfeldt C. Effect of vaccination on cattle subclinically infected with foot-and-mouth disease virus in Cameroon. Prev Vet Med. (2018) 155:1–10. 10.1016/j.prevetmed.2018.04.003 29786519

[38] Knowles NJ Wadsworth J Bachanek-Bankowska K King DP. VP1 sequencing protocol for foot and mouth disease virus molecular epidemiology. Rev Sci Tech (2016) 35:741–55. 10.20506/rst.35.3.2565 28332654

[39] Edgar RC. MUSCLE: multiple sequence alignment with high accuracy and high throughput. Nucleic Acids Res. (2004) 32:1792–7. 10.1093/nar/gkh340 15034147 PMC390337

[40] Kumar S Stecher G Tamura K. MEGA7: molecular evolutionary genetics analysis version 7.0 for bigger datasets. Mol Biol Evol. (2016) 33:1870–4. 10.1093/molbev/msw054 27004904 PMC8210823

[41] McDonald JH Handbook of Biological Statistics, 3rd ed. Baltimore, MD: Sparky House Publishing (2014).

[42] Balinda SN Sangula AK Heller R Muwanika VB Belsham GJ Masembe C. Diversity and transboundary mobility of serotype O foot-and-mouth disease virus in East Africa: Implications for vaccination policies. Infect Genet Evol. (2010) 10:1058–65. 10.1016/j.meegid.2010.06.017 20619358

[43] Jamal SM Ferrari G Ahmed S Normann P Curry S Belsham GJ. Evolutionary analysis of serotype A foot-and-mouth disease viruses circulating in Pakistan and Afghanistan during 2002–2009. J Gen Virol. (2011) 92:2849–64. 10.1099/vir.0.035626-0 21813704

[44] Hall MD Knowles NJ Wadsworth J Rambaut A Woolhouse MEJ. Reconstructing geographical movements and host species transitions of foot-and-mouth disease virus serotype SAT 2. MBio (2013) 4:e00591–13. 10.1128/mBio.00591-13 24149511 PMC3812709

[45] Kim H Xiao N Moritz M Garabed R Pomeroy LW Simulating the transmission of foot-and-mouth disease among mobile herds in the far north region, Cameroon. J Artific Soc Soc Simul. (2016) 19:6 10.18564/jasss.3064

[46] WRLFMD FMDV genotyping Africa (2015). Available online at: http://www.wrlfmd.org/west-africa/cameroon#panel-4952 (Accessed January 1, 2015).

[47] Ehizibolo DO Haegeman A De Vleeschauwer AR Umoh JU Kazeem HM Okolocha EC. Detection and molecular characterization of foot and mouth disease viruses from outbreaks in some states of Northern Nigeria 2013-2015. Transbound Emerg Dis. (2017) 64:1979–90. 10.1111/tbed.12602 28097814

[48] Bromham L Penny D. The modern molecular clock. Nat Rev Genet. (2003) 4:216–24. 10.1038/nrg1020 12610526

[49] Pybus OG. Model Selection and the Molecular Clock. PLOS Biol. (2006) 4:e151. 10.1371/journal.pbio.0040151 16683863 PMC1459243

[50] Brito BP Mohapatra JK Subramaniam S Pattnaik B Rodriguez LL Moore BR. Dynamics of widespread foot-and-mouth disease virus serotypes A, O and Asia-1 in southern Asia: a Bayesian phylogenetic perspective. Transbound Emerg Dis. (2018) 65:696–710. 10.1111/tbed.12791 29250910

[51] Carroll MW Matthews DA Hiscox JA Elmore MJ Pollakis G Rambaut A. Temporal and spatial analysis of the 2014–2015 Ebola virus outbreak in West Africa. Nature (2015) 524:97. 10.1038/nature14594 26083749 PMC10601607

[52] Real LA Henderson JC Biek R Snaman J Jack TL Childs JE. Unifying the spatial population dynamics and molecular evolution of epidemic rabies virus. Proc Natl Acad Sci USA. (2005) 102:12107–11. 10.1073/pnas.0500057102 16103358 PMC1186024

[53] Ahmed HA Salem SAH Habashi AR Arafa AA Aggour MGA Salem GH. Emergence of foot-and-mouth disease virus SAT 2 in Egypt during 2012. Transbound Emerg Dis. (2012) 59:476–81. 10.1111/tbed.12015 23025522

[54] Olabode OH Kazeem HM Raji MA Ibrahim NDG Foot and mouth disease in Nigeria - The current status and control efforts. Int J Livestock Res. (2014) 4:11–7. 10.5455/ijlr.20140514092453

