# Supplementary material for: Molecular Epidemiology of Foot-and-Mouth Disease Virus in the Context of Transboundary Animal Movement in the Far North Region of Cameroon
Source: Front Vet Sci. 2018 Dec 14;5:320. doi: 10.3389/fvets.2018.00320 (PMC6301994; doi:10.3389/fvets.2018.00320)
Supplement: Table S1 — Internal sequencing primers used to obtain the complete Cameroonian VP1 sequences. [file Data_Sheet_1.PDF]

| Target | In house Primer Name | Publication Primer Name | Sense | Sequence (5' TO 3') |
|---|---|---|---|---|
| FMD | Universal Forward | Universal Forward | forward | TGGTGACAGGCTAAGGATG |
| FMD | Universal Reverse | Universal Reverse | reverse | GCCCRGGGTTGGACTC |
| | | | | |
| Type O | CAM TYPE O F1 | CAR TYPE O F1 | forward | CGACTCAAAGGAGCCGGGCAA |
| Type O | CAM TYPE O F2 | CAR TYPE O F2 | forward | GGCGTCAACCGCTACGACCAG |
| Type O | CAM TYPE O F3 | CAR TYPE O F3 | forward | GCTGACGGCGATGCTTTGGT |
| Type O | CAM TYPE O 4F | CAR TYPE O 4F | forward | AAGCACCACTCAGAACAACG |
| Type O | CAM TYPE O R1 | CAR TYPE O R1 | reverse | TTCACTGCCACTTCTAGATCTG |
| Type O | CAM TYPE O R2 | CAR TYPE O R2 | reverse | TCGCCCGCAACGTGTACGTTG |
| Type O | CAM TYPE O R3 | CAR TYPE O R3 | reverse | TTGCCCGGCTCCTTTGAGTCG |
| Type O | CAM TYPE O 4R | CAR TYPE O 4R | reverse | GGCAAACCCAACCCTGTACG |
| SAT2 | CAM SAT2 F1 | CAR SAT2 F1 | forward | GCACGCTTTACGTGTTGGAGC |
| SAT2 | CAM SAT2 F2 | CAR SAT2 F2 | forward | CGGTGACAAGGTGATGGCAAC |
| SAT2 | CAM SAT2 F3 | CAR SAT2 F3 | forward | TGACCGCAGTACACATGTCC |
| SAT2 | CAM SAT2 4F | CAR SAT2 4F | forward | ACGTGAAGGGAGCCGGGCAG |
| SAT2 | PRIMER CAR SAT2 5F | CAR SAT2 5F | forward | AAGTTCATGGTGGCATACGTG |
| SAT2 | CAM SAT2 R1 | CAR SAT2 R1 | reverse | GGACATGTGTACTGCGGTCA |
| SAT2 | CAM SAT2 R2 | CAR SAT2 R2 | reverse | CACACCCAGGTATGGAACCTG |
| SAT2 | CAM SAT2 R3 | CAR SAT2 R3 | reverse | GGAACCACCACTAATGGCGT |
| SAT2 | CAM SAT2 4R | CAR SAT2 4R | reverse | GTCCCATTCCGCATGATAGC |
| SAT2 | PRIMER CAR SAT2 5R | CAR SAT2 5R | reverse | TTCGGTAGTAAACGTCGACT |
| Type A | PRIMER CAR TYPE A 1F | CAR TYPE A 1F | forward | AGTGCGCGTGAAAAATACCAG |
| Type A | PRIMER CAR TYPE A 1R | CAR TYPE A 1R | reverse | AGCTGTTTTGCGGGTGCAATG |
